# Chikungunya Virus: Priority Pathogen or Passing Trend?

**DOI:** 10.3390/vaccines11030568

**Published:** 2023-03-01

**Authors:** Gerardo Montalvo Zurbia-Flores, Arturo Reyes-Sandoval, Young Chan Kim

**Affiliations:** 1 The Jenner Institute, Nuffield Department of Medicine, University of Oxford, Roosevelt Drive, Oxford OX3 7DG, UK; 2 Instituto Politécnico Nacional (IPN), Av. Luis Enrique Erro s/n, Unidad Adolfo López Mateos, Mexico City 07738, Mexico; 3 Division of Structural Biology, Wellcome Centre for Human Genetics, University of Oxford, Roosevelt Drive, Oxford OX3 7BN, UK

**Keywords:** *Alphavirus*, mosquitoes, Chikungunya virus (CHIKV), chikungunya vaccine, clinical trial, epidemiology

## Abstract

Chikungunya virus (CHIKV) is considered a priority pathogen and a major threat to global health. While CHIKV infections may be asymptomatic, symptomatic patients can develop chikungunya fever (CHIKF) characterized by severe arthralgia which often transitions into incapacitating arthritis that could last for years and lead to significant loss in health-related quality of life. Yet, Chikungunya fever (CHIKF) remains a neglected tropical disease due to its complex epidemiology and the misrepresentation of its incidence and disease burden worldwide. Transmitted to humans by infected *Aedes* mosquitoes, CHIKV has dramatically expanded its geographic distribution to over 100 countries, causing large-scale outbreaks around the world and putting more than half of the population of the world at risk of infection. More than 50 years have passed since the first CHIKV vaccine was reported to be in development. Despite this, there is no licensed vaccine or antiviral treatments against CHIKV to date. In this review, we highlight the clinical relevance of developing chikungunya vaccines by discussing the poor understanding of long-term disease burden in CHIKV endemic countries, the complexity of CHIKV epidemiological surveillance, and emphasising the impact of the global emergence of CHIKV infections. Additionally, our review focuses on the recent progress of chikungunya vaccines in development, providing insight into the most advanced vaccine candidates in the pipeline and the potential implications of their roll-out.

## 1. Introduction

The rapid development of vaccines against SARS-CoV-2 in 2020 demonstrated that the collaborative effort of the scientific community and funding institutions can circumvent the traditional slow pace of vaccine development [1]. Being produced in record time, the first SARS-CoV-2 vaccines only took 16 months to be licensed and be rolled-out in the general population [1]. However, vaccine development is not always straightforward. Chikungunya virus (CHIKV) is a mosquito-transmitted disease considered a priority pathogen of major concern by governments and healthcare organisations around the globe due its potential to cause incapacitating disease [2,3,4]. Furthermore, CHIKV has been reported to emerge, disperse, and result in extensive outbreaks since the 1950s, and yet no licensed vaccine is available [5,6,7,8,9,10,11,12,13,14]. More than 50 years have passed since the first vaccine candidates against this virus were reported to be in development [15]. Notwithstanding the promising results of initial vaccine candidates, the development of CHIKV vaccines has been truncated by the limited availability of funding, the unpredictability of its epidemiology, and the fickle interest in this pathogen, which has gained and lost momentum as quickly as the CHIKV outbreaks have emerged and waned [15]. This review aims to highlight the importance of pursuing the development of a CHIKV vaccine until its licensure. In addition, this review emphasises the misrepresentation of the impact of CHIKV infections on global health. Furthermore, this review provides a brief update on the progression of those vaccine candidates advanced on the pipeline and the potential implications of their roll-out.

## 2. Chikungunya Virus (CHIKV)

### 2.1. Chikungunya Fever: An Incapacitating Disease

CHIKV is an arthropod-borne alphavirus from the *Togaviridae* family transmitted to humans by infected *Aedes* mosquitoes (*Aedes aegyti* and *Aedes albopictus* in urban cycles, and *Aedes furcifer* and *Aedes africanus* in sylvatic cycles) [6,7,10,11,16,17,18]. CHIKV is a small single-stranded positive-sense RNA virus with a genome consisting of 11.8Kb organised in two open-reading frames (ORF), one encoding four non-structural proteins (nsP1, nsP2, nsP3, and nsP4), and a second encoding five structural proteins including the capsid (C), peptide 6K/TF, and three envelope proteins (E1, E2, and E3) [5,16,18,19,20]. Although CHIKV is phylogenetically organised into four main lineages (West African, East/Central/South African-ECSA, Asian, and Indian Ocean Lineage-IOL), all CHIKV have the potential to cause chikungunya fever (CHIKF) in humans [8,10,15,20].

While CHIKV infections may be asymptomatic (up to 28%), they usually result in non-fatal and self-limiting acute fever illness along with other common symptoms including muscle pain, joint swelling, headache, nausea, rash, and fatigue [10,11,12,15,17]. Being the hallmark of CHIKF, symptomatic cases often report the development of severe arthralgia, which often transitions into incapacitating arthritis that lasts from months to years [11,15,17,20]. When CHIKF progresses to chronic CHIKF, convalescent individuals develop persistent chronic polyarthralgia, a debilitating condition that not only severely impacts the patient’s mobility but also their well-being, quality of life, and ability to perform day-to-day tasks [11,15,21,22,23]. Data from the extensive outbreak from la Reunion in 2004 suggests that up to 60% of individuals infected with CHIKV develop chronic Chikungunya [11]. Additionally, CHIKF has been reported to progress into severe acute Chikungunya fever (SA-CHIKF), occasionally causing deaths [7,11]. Observed mainly in infected children, elderly, immunosuppressed patients, or individuals with co-morbidities, SA-CHIKF can cause myocarditis, hepatitis, renal failure, and neurological complications such as encephalitis, myelopathy, peripheral neuropathy, or Guillain-Barré Syndrome [6,7,11,21,22,23]. Furthermore, CHIKV infection during pregnancy has been reported to cause congenital disease with teratogenic consequences, cause neonatal disease resulting in encephalitis, respiratory failure and delayed neurological development in infected children, or pregnancy termination [12,21,22].

### 2.2. Still Neglected: Underreported and Undiagnosed

According to the Pan American Health Organisation (PAHO) the number of reported CHIKV infections in the Americas decreased from over 2.5 million cases between 2013 and 2017 to under 800,000 cases between 2018 and 2022 [24]. However, this data must be treated with caution for two main reasons: the complexity of CHIKF diagnosis and the possible inaccuracy of the epidemiological data available. The epidemiological surveillance of CHIKV infection has always faced important challenges [25,26,27]. For instance, the difficulty of discriminating between CHIKF and other arbovirus-related illnesses due to the similarity of their clinical manifestations or possible co-infections [25,26,27,28,29]. A study investigating the diagnosis of dengue virus (DENV), Zika virus (ZIKV), and CHIKV in Nicaragua (2015–2016) reported that only 41.2% and 66.7% of patients with CHIKV and DENV infections, respectively, were accurately diagnosed based on clinical manifestations [30]. 

The co-circulation of CHIKV with other mosquito-borne viruses has been reported in Africa, the Americas, and the Pacific islands [30,31,32,33,34,35]. In the Pacific islands, concurrent circulation of DENV, ZIKV, and CHIKV has been extensively documented [33]. In fact, at least twenty outbreaks (11 DENV, 6 CHIKV, and 3 ZIKV) were documented to overlap between 2013 and 2014 [33]. Even though the emergence of their concurrent outbreaks poses an additional challenge for their diagnosis and aggravates their burden on healthcare systems worldwide, there is very little information about the prevalence of their co-circulation or co-infection [31,32,33].

Accurate diagnosis of CHIKF can only be confirmed by using specific serological and molecular tests that measure either the presence of anti-CHIKV antibodies in convalescent patients’ serum or the presence of CHIKV’s viral RNA [26,27]. Nevertheless, despite the known co-circulation of DENV, ZIKV, and CHIKV, individuals presenting “dengue fever-like” symptoms are not often tested against all three arboviruses. The requirement of specific testing for its diagnosis has led to the underreporting of CHIKF. In particular, the limited availability of resources in lower middle-income countries (LMICs), where CHIKV is more abundant, might prevent the confirmation of suspected cases of CHIKF. For instance, patients displaying fever-like-illnesses in the disadvantaged regions of the north of India have reportedly been tested only for dengue due to its higher incidence and mortality in the region [28]. Therefore, this could have resulted in the underreporting of other arboviral infections like CHIKV. A study investigating the prevalence of DENV, ZIKV, and CHIKV co-circulation and co-infection in Colombia highlighted the critical epidemiological situation of the Americas and the need to implement multiple-pathogen testing in regions were several mosquito-borne viruses co-circulate [32]. This study reported that the attack rates of DENV, ZIKV, and CHIKV were of 40.99, 58.38, and 29.72 cases per 100,000 inhabitants, respectively, between December 2015 and February 2016 [32]. Additionally, this study reported that co-infection of CHIKV with either DENV or ZIKV was observed in 7.64% and 5.10% of the confirmed cases, with an attack rate of 14.9 and 9.93 cases per 100,000 people, respectively [32]. Although the proportion of co-infections in the Colombian study is suggested to be low, other studies have indicated that the prevalence of co-infections might be higher. For instance, Waggoner et al. reported that more than a quarter of the infected patients in the study’s cohort suffered from co-infections with at least two or more of these viruses, which highlights the need for accurate, multiplex diagnostic methods against all three arboviruses for patient care and epidemiologic surveillance [30].

The underreporting of CHIKV infections is further highlighted by evidence indicating that, out of all suspected cases of CHIKV infection in the Americas (2018–2022), only 45–50% of reported cases are confirmed [24]. A great variability in reporting between countries further magnifies the misleading potential of some epidemiological data. For example, the epidemiological data provided by the PAHO from 2013–2017 and 2018–2022 indicates an overall decrease in reported cases of CHIKF in the Americas [24]. Nevertheless, the reported cases from 2013–2017 include the epidemiological data of all 52 countries in the region [24]. In contrast, the datasets from 2018–2022 failed to report the cases of CHIKV infection from almost half of the countries in the region [24]. Although the vast variability of reporting might have been caused by the focus of healthcare institutions around the world on the emergence and control of SARS-CoV-2 during this time period, the missing data suggests a decline in CHIKV infections when in fact the evidence from the same dataset indicates that CHIKV remains a cause of concern. Even when the number of cases of CHIKF in the Americas showed an apparent decrease between 2013–2017 and 2018–2022, the PAHO reported that the rate of CHIKV infections for 2021 was 500% greater than the one calculated for 2020, and alarmingly, 2021’s reported CHIKV infections had almost doubled by the 18th epidemiological week of 2022 [24]. Similarly, the World Health Organisation (WHO) reported an increase of cases of CHIKF in the West African countries of Ethiopia, Kenya, and Sudan towards the end of 2022 [36], and health authorities of India, Malaysia, Philippines, and Thailand reported an increase of CHIKV cases by the end of last year [36]. This overall rise in CHIKV cases highlights the relevance of improving the epidemiological surveillance of CHIKF. Given this continuous increase in CHIKV infections in the last decade, more emphasis should be placed on the need to pursue the development of intervention methods against CHIKV until their licensure for roll-out to the most vulnerable populations.

### 2.3. Chikungunya’s Burden: Significant but Poorly Documented

The global burden of CHIKV remains underestimated, and despite the evidence from the 2014 outbreak in Latin America suggesting that CHIKV could result in a greater burden than any other arthropod-borne virus [37], CHIKV is still considered a neglected tropical disease. The development of chronic arthritis is the most known long-term complication associated with CHIKV infection, and often the life-long chronic impairment caused by persistent polyarthralgia observed in previously infected patients is considered the only cause for decreasing an individual’s well-being and quality of life [38]. However, the global burden caused by CHIKV infection encompasses more than debilitating arthritis, and recently, other less-known clinical manifestations, complications, and long-term effects associated with its infection have been identified to contribute to the burden of CHIKF [38].

The first study calculating the burden of CHIKV in Latin America suggested that the outbreak in 2014 resulted in the loss of 151,031–167,950 disability-adjusted life-years (DALYs) [37]. Despite the limitations of this pioneer study, this evidence suggested that CHIKV’s burden was higher than that reported in previous epidemics [37]. Later, a study evaluating the global burden caused by CHIKV between 2010 and 2019 confirmed the potential high burden of CHIKV infection indicated by Cardona and collaborators [38]. This study also suggested that in average CHIKV causes a yearly loss of at least 158,000 DALYs, which according to the WHO, is just under the burden caused by DENV (203,000 DALYs) [38]. Lastly, Puntasseca and collaborators estimated that the chronic sequalae associated with CHIKV could result in between 401–7,977,910 years lived with disability (YLDs) [38].

In terms of economical loss, a study estimated that a CHIKV outbreak in the US Virgin Islands from 2014 to 2015 resulted in the loss $14.8–$33.4 million dollars [39]. The estimated loss considered both the direct and indirect costs of CHIKF. Whilst the direct cost of the illness included patient care, laboratory testing, prescription medication, and possible hospitalisation, the indirect costs considered losses after CHIKV infection including the average cost of absenteeism due to acute or long-term illnesses [39]. Although the limited availability of studies evaluating the burden of CHIKV infections is a huge limitation that must be recognised, the compatibility of the calculations between independent studies suggests possible plausibility. Overall, these studies provide substantial evidence showing that CHIKV still represents a major source of morbidity in CHIKV-endemic countries by contributing greatly to their long-term disease burden and resulting in substantial economic losses.

### 2.4. Here, There, and Everywhere: CHIKV’s Expanding Distribution

**Figure 1:**
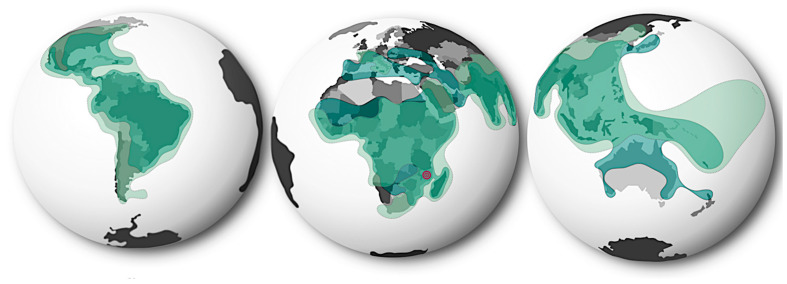
Overlapping distribution of the Chikungunya virus and the probability of occurrence of *Aedes* mosquitos (*A. aegypti* and *A. albopictus*). Originally identified in Tanzania in 1952 (red), CHIKV has been reported in over 100 countries in Africa, Asia, the Pacific, Oceania, the Americas, and the Caribbean (light green). The expansion of CHIKV’s geographical distribution across the globe has been perpetuated by the presence of *Aedes* mosquitos in non-endemic regions. The widespread distribution of CHIKV’s most common vectors (*A. aegypti* and *A. albopictus*) puts half of the world’s population at risk of infection (dark green).

Although it has been predicted that the increase of CHIKV infections in the next 20 years might be caused by the increase of human populations in CHIKV-endemic areas rather than to the further expansion of its geographical distribution, the possibility of CHIKV establishing in non-endemic regions remains a source of concern [26]. CHIKV has expanded its geographical spread dramatically since it was identified for the first time in Tanzania in 1952 [6,10,17,18]. Historically, CHIKV was contained in Africa, being maintained in enzootic reservoirs and only causing sporadic infections in humans [6,10,17,18]. Nevertheless, to date, more than 100 countries in the Americas, the Caribbean, North America, Western Pacific, Southern Europe, South-East Asia, and Oceania have reported the autochthonous transmission of CHIKV [5,6,10,12,17,18,19,40]. The early explosion of CHIKV’s distribution has been attributed to a single mutation (A226V) in the region of its genome encoding for the envelope protein 1 (E1) [7]. Occurring during the extensive outbreak in La Reunion in 2005, it is suggested that this mutation resulted in the adaptation of CHIKV to the *Aedes albopictus* mosquito, which enhanced its replication in this vector, and therefore led to increased viral transmission [7]. Added to possible mutations improving viral fitness and transmissibility, CHIKV’s geographical expansion may also have been accelerated by climate change and changes in vector and host populations [23,40,41,42]. Furthermore, it is undeniable that the globalisation of CHIKV has been driven by anthropogenic activity [10,23,43,44,45,46,47]. The explosion of human populations and changes in land use have contributed to the spill-over of CHIKV and have promoted the establishment of urban infection cycles [10,23,43,44,45,46]. Moreover, increased global interconnectivity, the increased trade of goods and animals, and increased human migration and tourism has facilitated the dispersion of CHIKV globally [10,23,43,44,45,46,47]. The continuous expansion of CHIKV’s geographical distribution, hand-in-hand with its vector’s increasing distribution, is a major concern (Figure 1) [25,27,48], particularly considering that, to date, *Aedes* mosquitos have the widest distribution ever reported, putting at risk of infection more than three billion people [27,48,49]. As climate change progresses and the temperatures in temperate regions increase, *Aedes* mosquitos establish populations in regions where they were not originally endemic [9,17,48,49]. Specifically, the distributions of *A. aegypti* and *A. albopictus* have been predicted to expand as the environmental suitability for them to thrive increases [9,17,48,49]. The possible expansion of the distribution of *A. aegypti* and *A. albopictus* into more temperate regions of the globe has further highlighted the increasing threat that CHIKV and other arboviruses pose to global health [26].

### 2.5. Chikungunya Vaccines on the Horizon

Epidemiological data suggesting that single CHIKV infection confers lifetime protection against all existing lineages suggests that vaccination may be sufficient to provide lifelong protection [6,15,18,50,51]. Therefore, vaccination remains the best strategy to control extensive CHIKV epidemics [6,18]. At least 30 vaccine candidates have been tested in preclinical settings [15,19,20,52,53,54,55,56,57,58,59,60,61,62,63,64,65,66,67,68,69,70,71,72,73,74,75], of which, five have progressed to Phase I clinical trials [50,51,76,77,78,79], two to Phase II clinical trials [80,81], and two to Phase III (Table 1). Yet no licensed vaccine is available against CHIKV to date [8,15,18,19,20].

As the history of CHIKV vaccines has been reviewed recently [7,11,15], we will only discuss the progress of those vaccines which are most advanced in the development pipeline. Most recently, a CHIKV vaccine candidate based on the same chimpanzee replication-deficient adenoviral vector used in the Oxford-AstraZeneca COVID-19 vaccine (AZD1222) has showed promising results [50,53,61,69]. The ChAdOx1-CHIK encodes the CHIKV’s structural genes (Capsid, E1, E2, E3 and peptide 6K/TF) of a mosaic consensus sequence derived from multiple CHIKV lineages (Asian, ECSA, and West African) [61]. The safety and immunogenicity of ChAdOx1-CHIK was evaluated in a dose-escalation Phase I clinical trial (NCT03590392) in healthy adults, in which ChAdOx1-CHIK demonstrated the achievement of complete seroconversion of vaccine recipients after a single unadjuvanted dose [50,82]. Additionally, ChAdOx1-CHIK induced broadly neutralising antibodies against four CHIKV lineages (Indian Ocean, West African, Asian, and Asian-American) in all participants and as early as 2 weeks after vaccination [50]. As ChAdOx1-CHIKV showed excellent safety, tolerability and 100% PRNT_50_ seroconversion after a single unadjuvanted dose in Phase I clinical trial, ChAdOx1-CHIK soon entered a Phase 1b clinical trial (NCT04440774) in order to evaluate its safety and immunogenicity when administered alone or in co-administration with ChAdOx1-ZIKV in healthy adults in Mexico [19]. This study was completed in March of 2022, and the outcome of this trial is yet to be published [19]. 

One of the three vaccine candidates that have reached Phase II clinical trials is MV-CHIK, a recombinant Measles virus vector encoding the structural polyprotein genes (Capsid, E3, E2, 6K/TF, and E1) of CHIKV’s Indian Ocean lineage [52]. When assessed in a dose-escalation Phase I clinical trial (NCT03028441), MV-CHIK showed promising results with a good safety profile [83]. Although total seroconversion was achieved only after a homologous prime-boost, a single immunisation induced highly neutralising antibodies with intermediate (7.5 × 10^4^ TCID_50_) and high doses (3.0 × 10^5^ TCID_50_) [79]. Similarly, a follow-up double-blind, randomised, placebo-controlled and active-controlled trial (NCT02861586) evaluating the immunisation interval (28 or 168 days) between homologous MV-CHIK prime-boost demonstrated good tolerability whilst inducing robust neutralising antibodies [81,84]. 

One step further in the pipeline, the VRC-CHKVLP059-00-VP is currently being evaluated in Phase III clinical trials (NCT05072080 and NCT05349617) [85,86]. VRC-CHKVLP059-00-VP is a vaccine based on the immunisation of CHIKV virus-like particles containing all of the structural proteins (Capsid, E3, E2, 6K/TF, and E1) encoded by the structural polyprotein genes of the West African lineage [51]. Evaluation of VRC-CHKVLP059-00-VP in Phase I clinical trials demonstrated to be highly immunogenic after a single immunisation whilst remaining well tolerated [87,88]. VRC-CHKVLP059-00-VP induced highly neutralising antibodies in most vaccinees after a single dose and in all participants four weeks after a homologous boost [88]. Having entered Phase II clinical trials in 2015, the VRC-CHKVLP059-00-VP was evaluated amongst 400 healthy adults in multiple locations on the globe (NCT02562482) [87,89]. Later known as PXVX0317, the VRC-CHKVLP059-00-VP was evaluated in a Phase II clinical trial in order to investigate its safety and immunogenicity in response to different doses, vaccination schedules, and formulations, including its co-administration with aluminium hydroxide as adjuvant (NCT03483961) [89]. Overall, PXVX0317 was well tolerated and induced robust long-lasting neutralising antibody responses in prime-boost regimens [90]. A homologous prime-boost regimen (20 µg/dose) separated by 28 days induced the highest titre of neutralising antibodies with detectable immune responses up to two years [90]. Interestingly, the other subject groups which showed the longest durability of responses (up to two years) were those receiving single unadjuvanted high doses (40 µL) or two unadjuvanted standard doses (20 µL) [90]. Ultimately, the induction of a rapid and robust immune response was prioritized [90]. As the use of aluminium hydroxide elicited neutralising antibody responses earlier than unadjuvanted doses [85], PXVX0317 (adjuvanted with aluminium hydroxide) is being evaluated in healthy adolescents, adults, and older adults (>65) in the ongoing Phase III clinical trials (NCT05072080 and NCT05349617) [85,86].

Another CHIKV vaccine ongoing Phase III clinical trials is the VLA 1553 (Valneva), a live-attenuated vaccine based on a genetically engineered strain of CHIKV of the ECSA lineage with a deletion in the nsP3 gene, which induces attenuation [78]. In a dose-escalation Phase I clinical trial (NCT03382964) completed in July 2009, a single immunisation with VLA 1553 vaccine induced complete seroconversion of vaccine recipients and was well tolerated in all dose groups [78,91]. Immune responses induced by VLA 1553 were also demonstrated to be maintained for at least 12 months in all groups [78]. According to Katrin Dubrischar, VP and Program Director of Valneva’s CHIKV vaccine, as a single dose of VLA 1553 induced antibody levels that reached plateau in all dose groups, no Phase II clinical trial was deemed necessary [92]. Therefore, in March 2020 Valneva announced the progression of the VLA 1553 to Phase III [3]. Since then, VLA 1553 started a series of Phase III clinical trials of which two are completed (NCT04786444 and NCT04546724) [93,94]. The first Phase III trial involved a randomised, double-blinded study to investigate lot-to-lot manufacturing consistency in over 400 subjects [88]. Despite the study’s completion date in January 2022, the quality control review process has not concluded [95]. The second trial, which has been completed, evaluated the final dose of VLA 1553 (1 × 10^4^ TCID_50_ per dose) with a placebo control in over 4000 adults [94]. This study confirmed the robust immunogenicity of VLA 1553, which induced titres of neutralising antibodies with an average of over 3200 units and resulted in an overall seroconversion rate of 98% among participants 29 days after immunization [94]. Two more Phase III clinical trials for VLA 1553 are currently recruiting. The first one is a study to evaluate the final vaccine dose in adolescents (NCT04650399), and the latter will comprise a Phase IIIb clinical study to assess the long-lasting persistence of antibodies and long-term safety of vaccine recipients (NCT04838444) [96,97]. The completion of both studies is expected by March 2024 and December 2025, respectively [96,97].

The potency and safety of Valneva’s CHIKV vaccine might provide to the world an invaluable tool to combat CHIKV epidemics. Furthermore, the licensure of one CHIKV vaccine should encourage the progression of the others in order to minimise the burden of CHIKV and its associated diseases to the furthest extent. Though promising, the possible success of any of these CHIKV vaccines must take into account two major considerations: first, the licensed vaccines must be able to provide protection against heterologous lineages [15]. This is particularly relevant considering that most vaccine candidates in advanced stages of development are based on sequences from different lineages [50]. Moreover, the efficacy of licensed vaccines will have to be monitored closely after their approval, as novel strains of CHIKV have rapidly emerged during extensive outbreak scenarios [13,14]. Second, a series of logistical considerations related to vaccine equity and the overall expenses of vaccine roll-out must be contemplated in order to fully control CHIKV epidemics; especially considering that CHIKV is most abundant in regions with limited resources such as LMICs. In this regard, Valneva has reportedly made an agreement with the Coalition for Epidemic Preparedness Innovations (CEPI) committing to provide equitable access to the CHIKV vaccine to all LMICs [3]. Valneva has also agreed to maintain a vaccine stockpile and to extend the equitable access to CHIKV vaccines to those countries at risk or under extensive outbreak scenarios [3].

## 3. Concluding Remarks

CHIKV remains a major unmet medical need. Like most tropical diseases, CHIKV is often underreported, undiagnosed, and underestimated, predominantly impacting regions with limited resources. Therefore, the present review emphasises the potential threat that CHIKV poses to global health by providing evidence of its estimated disease and economic burden. This review also highlights the need to develop intervention methods against neglected tropical diseases, such as CHIKV, whose clinical importance has been depreciated by factors rooted in their epidemiological complexity, and the incidence of which has been misrepresented due to the socio-economical limitations of the regions where they are endemic. Furthermore, this review highlights the possible expansion of CHIKV’s geographical distribution and its potential to rapidly disperse among naïve populations. Lastly, this review provided an encouraging overview of the most advanced CHIKV vaccines to date, which may be available sooner than we think.

## Figures and Tables

**Table 1:** General features of CHIKV vaccines that have entered clinical trials (Phase I/II/III).

Vaccine Candidate	Vaccine Platform	Lineage	Strategy	Manufacturer	Stage of Development
CHIK Vaccine FIV 15562	Inactivated	Asian	Formalin virus inactivation.	US WRAIR	Phase I
CHIK ECSA	Inactivated	Indian Ocean	Formalin or beta-propiolactone virus inactivation.	Bharat Biotech International Limited	Phase I
CHIK IRES	Live-attenuated	Indian Ocean	Attenuation through the addition of EMCV’s internal ribosome entre sequence (IRES) to encode CHIKV structural proteins.	Takeda	Phase I
mRNA-1388 (VAL-181288)	mRNA	-	Encodes CHIKV structural proteins	Moderna	Phase I
ChAdOx1 CHIKV	Viral Vector	Multi- lineage	Mosaic consensus sequences encoding the whole structural polyprotein (Capsid, E1, E2, E3, and 6K).	Jenner Institute, University of Oxford	Phase Ib
CHIKV TSI-GSD-218	Live-attenuated	Asian	Attenuation in green monkey kidney cells (GMKC) and in human embryonic lung MRC-5 cells.	US Army MRIID	Phase II (no further progression)
MV-CHIK	Viral Vector	Indian Ocean	Recombinant Measles vector encoding CHIKV’s structural polyprotein (Capsid, E1, E2, E3, and 6K).	Institut Pasteur & Themis Bioscience GmbH	Phase II
VRC-CHKVLP059-00-VP	Virus-like-particle (VLP)	African	Expression of structural proteins (Capsid, E1, E2, E3, and 6K) in vitro to produce particles as empty shells.	US NIH’s NIAID	Phase II, progressed as PXVX0317
PXVX0317	Virus-like-particle (VLP)	African	VRC-CHKVLP059-00-VP mixed with aluminium hydroxide adjuvant.	US NIH’s NIAID	Phase III
VLA1553-302	Live-attenuated	ECSA	Attenuation by Δ5NS3 gene deletion	Valneva & Butantan	Phase III
